# Multi-Objective Whale Optimization Algorithm for Computation Offloading Optimization in Mobile Edge Computing

**DOI:** 10.3390/s21082628

**Published:** 2021-04-08

**Authors:** Mengxing Huang, Qianhao Zhai, Yinjie Chen, Siling Feng, Feng Shu

**Affiliations:** 1 School of Information and Communication Engineering, Hainan University, No. 58 Renmin Avenue, Haikou 570228, China; huangmx09@hainanu.edu.cn (M.H.); chenyinjie@hainanu.edu.cn (Y.C.); shufeng@hainanu.edu.cn (F.S.); 2 State Key Laboratory of Marine Resource Utilization in the South China Sea, Hainan University, No. 58 Renmin Avenue, Haikou 570228, China; 3 School of Sciences, Hainan University, No. 58 Renmin Avenue, Haikou 570228, China; zhaiqh@hainanu.edu.cn

**Keywords:** edge computing, computation offloading, multi-objective, whale optimization algorithm

## Abstract

Computation offloading is one of the most important problems in edge computing. Devices can transmit computation tasks to servers to be executed through computation offloading. However, not all the computation tasks can be offloaded to servers with the limitation of network conditions. Therefore, it is very important to decide quickly how many tasks should be executed on servers and how many should be executed locally. Only computation tasks that are properly offloaded can improve the Quality of Service (QoS). Some existing methods only focus on a single objection, and of the others some have high computational complexity. There still have no method that could balance the targets and complexity for universal application. In this study, a Multi-Objective Whale Optimization Algorithm (MOWOA) based on time and energy consumption is proposed to solve the optimal offloading mechanism of computation offloading in mobile edge computing. It is the first time that MOWOA has been applied in this area. For improving the quality of the solution set, crowding degrees are introduced and all solutions are sorted by crowding degrees. Additionally, an improved MOWOA (MOWOA2) by using the gravity reference point method is proposed to obtain better diversity of the solution set. Compared with some typical approaches, such as the Grid-Based Evolutionary Algorithm (GrEA), Cluster-Gradient-based Artificial Immune System Algorithm (CGbAIS), Non-dominated Sorting Genetic Algorithm III (NSGA-III), etc., the MOWOA2 performs better in terms of the quality of the final solutions.

## 1. Introduction

With the rapid development of technologies, terminal devices, especially Internet of Things (IoT) devices and smartphones, are limited in resources, while applications running on them are resource-hungry. In this case, cloud computing allows data from terminal devices to be processed at servers through the Internet, relieving computation pressure at the terminal devices [1]. Unfortunately, some applications are latency-sensitive and compute-intensive; cloud computing is not suitable for these applications [2]. As a supplement of cloud computing, edge computing deploys small-size cloud-computing-like capabilities at the edge of the network [3]. The main difference between these two technologies is reflected in the physical location where storing and processing are performed [4]. Edge computing turns centralized clouds into distributed pervasive fogs. Terminal devices can send data to the closest server with lower latency and lower power consumption. Moreover, edge computing has better cognition [5], agility and security [6]. Additionally, the combination of 5G technology and edge computing produces new developments in many areas, such as smart robots and smart farming [7].

Computation offloading plays an essential role in edge computing. It is firstly proceeded in cloud computing and then used in edge computing. Devices can transmit computation tasks to servers to be executed through computation offloading. Additionally, not all the computation tasks can be offloaded to servers in common cases with the limitation of network conditions [8]. Therefore, it should be quickly decided how many tasks should be executed on servers and how many should be executed locally. Only properly offloaded computation tasks can achieve the Quality of Service and increase the Quality of Experience.

Optimizing computation offloading is typically formulated as a Mixed Integer Non-Linear Programming (MINLP) problem, which is challenging to solve. The optimizing approaches of computation offloading in mobile edge computing are separated into several perspectives: game-theoretic methodology [9], machine learning methodology [10], queuing theory, and linear programming concerning other algorithms. Researchers are trying to solve computation offloading problems using various methods. Among these, game-theoretic methodology and machine learning methodology are two major perspectives. Many excellent works have been studied regarding the computation offloading problem. For example, Gianni and Palmieri et al. [11] proposed a modified genetic algorithm for local searching, which shows quite good results; Gianni and Tipaldi et al. [12] use rule-based machine learning to optimize Markov decision process modelled spacecraft autonomy, which also achieves effectiveness. Both the game-theoretic methodology and machine learning methodology are reasonable and possible.

For the game-theoretic methodology, using game-theoretic approaches to determine pure Nash equilibria is an efficient non-deterministic approach, and game-theoretic-based techniques are widely utilized in this field. Chen et al. [13] jointly formulated the computation offloading problem and solved it using a game-theoretic approach by showing the existence of a Nash equilibrium; Wang et al. [14] designed a partial computation offloading method that optimized both communication and resources; Ma et al. [15] proposed an energy-aware computation offloading algorithm; Dong et al. [16] proposed an evolutionary game approach to optimize the task offloading in edge computing; Elgendy et al. [17] proposed an efficient offloading algorithm achieving the computation offloading decision for computation tasks, which use the method of finding the near-optimal computation offloading and compression decision; Zhou and Jadoon [18] proposed a partial computation offloading strategy based on game theory for multi-user edge computing; Wang et al. [19] proposed a decentralized computation offloading algorithm with multi-agent imitation learning.

For the machine learning methodology, approaches based on machine learning have also been used in recent years for more and more pervasive devices which have appeared. It is effective and practical but may have higher complexity than game-theoretic approaches and need more resources, which mean this mechanism can be used on inherently limited devices. Hossain et al. [20] presented an optimal binary computational offloading decision using reinforcement learning; Huang et al. [21] investigate low-complexity computation offloading strategies to minimize energy consumption and propose a distributed deep learning-based offloading algorithm to achieve better convergence; Zhang et al. [22] evaluate an online learning offloading framework for heterogeneous mobile edge computing by conducting a failure recovery policy; Xie et al. [23] minimize service time by dividing tasks into subtasks and apply the method proposed to the computation offloading problem among vehicles; Cui et al. [24] use stochastic online learning that can learn from the changes of dynamic systems, and the method is used for the distributed system; Xu et al. [25] design an energy-aware computation offloading method to reduce the energy consumption and adopt a Non-dominated Sorting Genetic Algorithm II (NGSA-II) to shorten the offloading time of the computing tasks.

To clarify the effectiveness of these works. The advantages and weakness of these works mentioned above are listed in Table 1.

It can be seen from Table 1 that the computation offloading optimization problem can be solved in many aspects. Some methods have low complexity and easy to apply but only have one target; some other methods are comprehensive but show high complexity and are hard to implement. Finding a solution to strike a balance is needed, including key targets in computation offloading optimization problems with reasonable complexity. The method proposed in this not only paper takes multiple objections into consideration but also has obvious advantages in convergence, diversity and complexity.

The Whale Optimization Algorithm (WOA) is an optimization algorithm proposed by Australian scholars Mirjalili and Lewis in 2016 [26]. The WOA has promising advantages. It is insensitive to the initial solutions, which may have a significant influence on some traditional algorithms. Additionally, it has adaptive mechanisms to balance the explorative and exploitative behaviors properly. It has been widely used in feature selection, parameter extraction, engineering optimization, and other aspects. It does not need to compute gradients, which is fit for computation offloading scenarios [27].

Tongbram et al. [28] introduce WOA into image segmentation with a noise detection and reduction mechanism. Hassouneh et al. [29] combine WOA with a single point crossover method and use the enhanced WOA to predict software faults. Abdel-Basset et al. [30] adapt WOA for a DNA fragment assembly problem. Shanky et al. [31] designed an energy resource allocation framework optimized based on WOA. As an expansion of WOA, the Multi-Objective Whale Optimization Algorithm (MOWOA) was proposed to solve optimization problems that have more than one target, inheriting the advantages of WOA. A distributed clustering algorithm using multi-objective whale optimization is proposed by Kotary et al. [32] for a peer-to-peer network. A multi-objective whale optimization algorithm proposed by Wang et al. [33] is used to solve the energy-efficient distributed permutation flow shop scheduling problem. Ehteram et al. [34] proposed a hybrid Artificial neural network (ANN) with a multi-objective whale optimization algorithm designed to perform suspended sediment load prediction.

WOA is introduced to solve computation offloading problems by Pham, Huong et al. [35] and Pham, Quoc et al. [36]. Pham, Huong et al. employ WOA to solve the Transmit Power Control (TPC) problems and they find the simplicity and efficiency of WOA on TPC problems. Pham, Quoc et al. apply WOA to resource allocation optimization problems and obtain a promising conclusion. However, MOWOA has still not been applied to solving the optimal offloading mechanism of the computation offloading in mobile edge computing. The motivation for proposing MOWOA and improved MOWOA for the computation offloading in this research is three-fold. First, the optimization problem in computation offloading is an important problem that needs to be overcome. As with some methods list in Table 1, the existing methods are not good enough or not practical. Finding a satisfying solution is apparently required. Second, the whale optimization algorithm is universal for many application areas with the advantages mentioned, and has many successful applications [28,29,30,31,32,33,34]. Third, WOA is used to solve the optimization strategy of computing offloading [35,36]. However, they only focus on just one factor and ignore the fact that the optimization of computation offloading may be affected by multiple factors. Last, considering that time consumption and energy consumption are the most important factors in the computation offloading optimization problem, MOWOA has the potential to solve the optimal offloading mechanism of computation offloading in mobile edge computing. Therefore, in this paper, MOWOA can be used to solve the problem mentioned.

The key contributions of this paper are as follows. Firstly, a method of using MOWOA to solve the optimal offloading mechanism of computation offloading in mobile edge computing is proposed. Secondly, the parameters of the algorithm are modified to suit our model. Furthermore, the gravity reference point method is employed to further improve MOWOA. The improved MOWOA is named MOWOA2. Finally, in order to improve the quality of the solution set, crowding degrees are introduced, which are defined as the summation of the ratio of the difference of the objective functions of two adjacent solutions with that of two extremum solutions; then, all solutions in the set are sorted in descending order by crowding degrees.

The paper is organized as follows. Section 2 shows the computation offloading model in edge computing. The MOWOA and MOWOA2 for solving the model are described in Section 3. Section 4 presents numerical experiments and analyzes the performance of our methods. Section 5 analyzes and discusses the performance of our methods. Finally, a brief conclusion is given in Section 6.

## 2. The Computation Offloading Model

In this section, there will be a detailed discussion of the computation offloading model. The edge computing system consists of an edge server and *n* mobile devices users with each task, denoted by a set N = {1, 2, …, n}. There is a wireless base-station *s*, through which the mobile device users can offload the computation to the edge computing servers. Next, the communication and computation models are introduced in detail, both of which play vital roles in mobile edge computing.

### 2.1. Communication Model

Firstly, the communication model with wireless access used in mobile edge computing is introduced. The wireless base-station *s* manages the communications of mobile device users. The set of wireless channels that *s* can use are denoted as Μ = {1, 2, 3, …, M}. Moreover, $X\;=\;\left(x_{1},\;x_{2},\;\ldots ,\;x_{n}\right)$ is a decision vector, where $x_{i}$(*i* = 1, 2, …, n) represents that mobile device user *i* offloads *x_i_* × 100% of its task to be executed on edge servers, and the rest $\left(1\;-\;x_{i}\right)\;\times \;100\%$ of its task to be executed locally. Such a kind of decision vector is used to overcome the problems and disadvantages of the computation offloading model that uses 0–1 planning. In the model, the transmission rate *r_i_* of user *i* is denoted as [37]

(1) $$r_{i}=\omega \log_{2}\left(1+\frac{p_{i}h_{i}}{\sigma^{2}-p_{i}h_{i}+\sum_{j=1}^{n}p_{j}h_{j}}\right)r_{i}=\omega \log_{2}\left(1+\frac{p_{i}h_{i}}{\sigma^{2}-p_{i}h_{i}+\sum_{j=1}^{n}p_{j}h_{j}}\right)$$

in which *ω* is the bandwidth of the channel, *p_i_* is the transmission power of user *i*, h*_i_* is the channel gain between user *i* and base-station *s*, and σ^2^ represents the background noise power. $-p_{i}h_{i}+\sum_{j=1}^{n}p_{j}h_{j}$ represents the effects from other mobile device users. From the communication model in (1) it can be determined that if too many mobile device users choose to offload the computation via the same channel concurrently, this may lead to severe interference and cause low data rates, which would negatively affect the performance of mobile edge computing.

### 2.2. Computation Model

Then, the computation model is introduced. The tasks set are defined as C = {c_1_, c_2_, …, c_n_}, in which c*_i_* represents the total number of Central Process Unit (CPU) cycles needed to finish the computation task of user *i*, and define the data size set B = {b_1_, b_2_, …, b_n_}, in which b*_i_* represents the data size of the task of user *i*. Next, the computation overhead in the field of both energy consumption and processing time for both local and remote computing approaches are discussed.

(1) Local Computing: For the local computing approach, a mobile device user n executes its computation task locally on the mobile device. The computation execution time $T_{i}^{L}$ of the task user *i* locally is given as

(2) $$T_{i}^{L}=\frac{u}{Z_{i}^{L}}$$

where $Z_{i}^{L}$ is the computation capability (i.e., CPU cycles per second) of the mobile device user *i*, $u=(1-x_{i})c_{i}$. $x_{i}$ is the decision vector mentioned in Section 2.1. The $x_{i}$ referred to below also has the same meaning. For the energy of the computation, it holds that

(3) $$E_{i}^{L}=\eta {\left(Z_{i}^{L}\right)}^{2}u$$

in which η is the coefficient of the consumed energy per CPU cycle, which can be obtained by the measurement method in [38] and it is set as 1 × 10^−26^.

(2) Remote Computing: for the remote computing approach, a mobile device user *i* offloads its computation task to the edge servers via wireless base-station *s*.

Using computation offloading would cause extra overhead in the fields of time and energy for transmitting the computation data. The total time overhead of mobile device user *i* for offloading the task to edge servers is computed as

(4) $$T_{i}^{M}=\;v+\frac{k}{Z_{i}{}^{M}}$$

in which $Z_{i}^{M}$ is the computation capability available for mobile device user *n*, and it is determined by the servers. It can be supposed that the computation capability of each user is equal, which means $Z_{1}^{M}=Z_{2}^{M}=\cdot \cdot \cdot =Z_{n}^{M}$. $v=\frac{x_{i}b_{i}}{r_{i}}$, $k=x_{i}c_{i}$. $b_{i}$ is the time used by user *i* to transmit data to servers and $\frac{k}{Z_{i}^{M}}$ is the execution time that servers need for the task from user *i*. Due to the fact that the data size of the computation results is usually much smaller than that of input data, the time overhead of sending the result back is neglected.

Then, the total energy overhead for mobile device user *i* to offload the task to edge servers is defined as

(5) $$E_{i}^{M}=p_{i}v+\eta {\left(Z_{i}^{M}\right)}^{2}k$$

where $p_{i}v$ is the energy consumption produced from user *i* to edge servers, during the data transmission, and $\eta {\left(Z_{i}^{M}\right)}^{2}k$ is the energy consumption that servers need to execute the task from user *i*.

Finally, the optimization model is defined as:

(6) $$\begin{array}{ll} \min & F(X)=(f_{1}(X),f_{2}(X))\\ & f_{1}(X)=\sum_{i=1}^{n}\left(T_{i}^{L}+T_{i}^{M}\right)\\ & f_{2}(X)=\sum_{i=1}^{n}\left(E_{i}^{L}+E_{i}^{M}\right)\\ s.t. & X\;=\;\left(x_{1},\;x_{2},\;\ldots ,\;x_{n}\right),\;x_{i}\in [0,1]\;,\;i=1,2,\ldots \ldots ,n \end{array}$$

where $f_{1}(X)$ is the total time for computing the tasks, $f_{2}(X)$ is the total energy consumption for those tasks, and *n* is the number of device users.

## 3. Multi-Objective Whale Optimization Algorithm (MOWOA) for Solving Model

### 3.1. Multi-Objective Optimization

The process of searching for the best solution or optimal value from an optimization problem is referred to as optimization. The target of optimization can be single or more. The optimization problems with more than one objective are called multi-objective optimization [39]. This kind of problem can be found almost everywhere, such as in mathematics, economics, engineering, computer science, etc. There are two kinds of methods commonly used: the Pareto Method [40] and the Scalarization Method [41]. For the Pareto Method, Pareto optimality is a situation where no individual or preference criterion can be better off without making at least one individual or preference criterion worse off or without any loss thereof. In this paper, a multi-objective method based on Pareto Method is used.

### 3.2. Whale Optimization Algorithm

**Figure 1:**
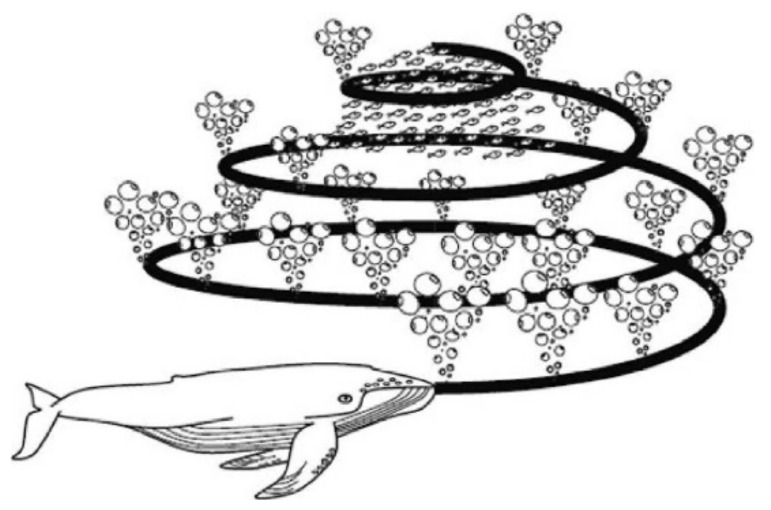
Bubble-net attacking behavior of humpback whales.

WOA is a swarm intelligence optimization algorithm, which is inspired by the unique hunting method of the humpback whales, which is called the bubble-net attacking approach shown in Figure 1.

**Figure 2:**
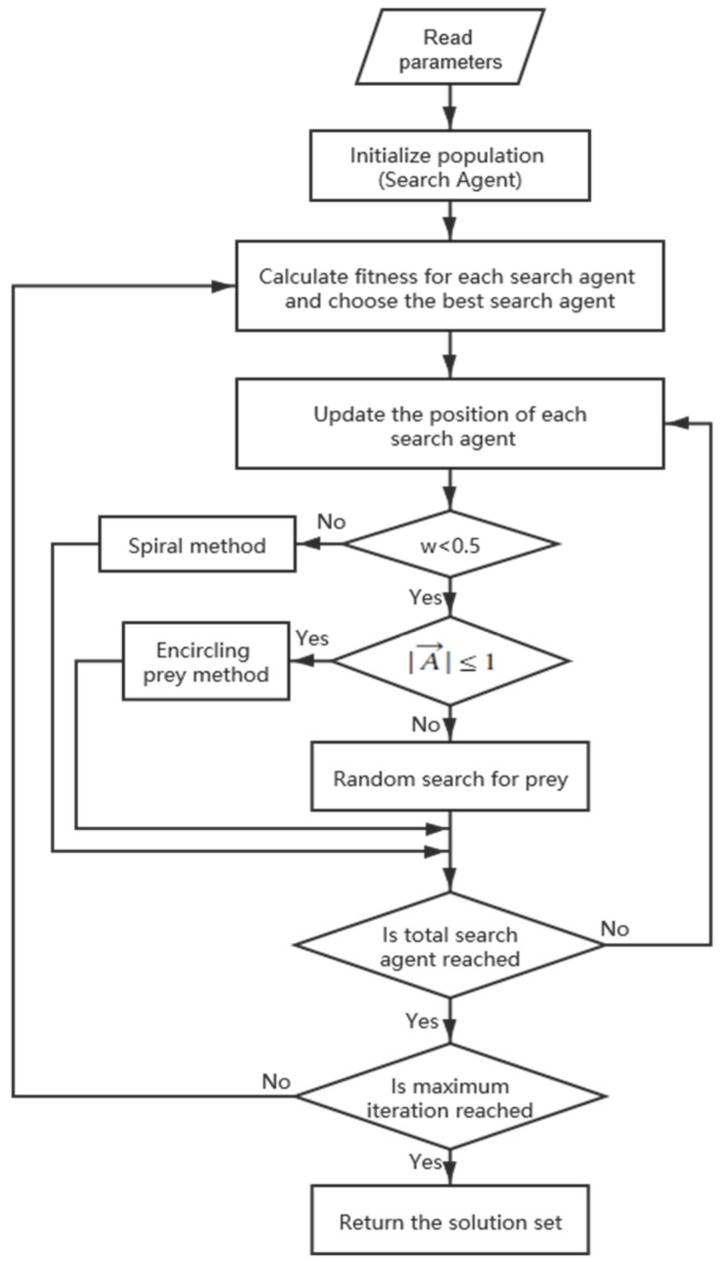
Flowchart of the whale optimization algorithm.

Groups of krill or small fishes are the preferred food of humpback whales. When humpback whales are hunting, they have two kinds of behaviors associated with a bubble-net [42]. One is named upward-spirals. They dive down and start to create bubbles in a spiral shape around the prey and swim up toward the surface; another is named double-loops with three stages: the coral loop, lobtail, and capture loop. The thought of WOA is to imitate the behaviors of humpback whales, and the flowchart of the method is shown in Figure 2, where w is a random number in [0,1] and $\vec{A}$ is a vector whose definition is on formula (7).

Before the search agent updates its position, $\vec{r}\in [0,1]$ is a randomly generated vector to calculate $\vec{A}$ and $\vec{C}$. The coefficient vector $\vec{A}$ and $\vec{C}$ are calculated as follows:

(7) $$\vec{A}=2\cdot \vec{a}\cdot \vec{r_{}}-\vec{a}$$

(8) $$\vec{C}=2\cdot \vec{r_{}}$$

where $\vec{a}$ is a vector that linearly decreased from two to zero in the iterations.

#### 3.2.1. Encircling Prey

If w<0.5 and $\left|\vec{A}\right|\leq 1$, the stage of encircling prey starts. Humpback whales can recognize the location of prey and encircle them. The current best candidate solution is supposed to the target prey or is close to the optimum. After the best search solution is defined, the other search solutions will hence try to update their positions towards the best search solution. This behavior is represented by the following equations:

(9) $$\vec{D}=\left|\vec{C}\times \vec{P_{*}^{t}}-\vec{P_{i}^{t}}\right|$$

(10) $$\vec{P_{i}^{t+1}}=\vec{P_{i}^{t}}-\vec{A}\times \vec{D}$$

where *t* indicates the current iteration, $\vec{P_{*}^{t}}$ is the position vector of the best solution obtained so far, $\vec{P_{i}^{t}}$ is the position vector, | | is the absolute value. $\vec{P_{*}^{t}}$ should be updated in each iteration if there is a better solution. 

#### 3.2.2. Bubble-Net Attacking 

If w≥0.5, the stage of bubble-net attacking starts. Inside the shrinking enclosure, humpbacks follow spiraling paths toward their prey, a method known as bubble-net foraging. Therefore, WOA calculates the distance between the whale and its prey when it uses the spiral method to update its position. In order to simulate the spiral motion of the humpback whale, the mathematical formula is as follows:

(11) $$\vec{P_{i}^{t+1}}=\vec{D}’\cdot e^{bl}\cdot \cos(2\pi l)+\vec{P_{*}^{t}}$$

where $\vec{D}=\left|\vec{P_{*}^{t}}-\vec{P_{i}^{t}}\right|$ indicates the distance of the *i* th whale to best solution obtained so far, *b* is a constant for defining the shape of the logarithmic spiral, *l* is a random number in [−1,1]. 

#### 3.2.3. Random Search for Prey

If w<0.5 and $\left|\vec{A}\right|>1$, the stage of a random search for prey starts. In the process of hunting prey, whales need to locate their prey. Once the location is established, the whales are able to encircle their prey. The current best search agent is assumed as the target prey; all the whales will update their position with the tendency of moving closer to the prey. This procedure will iterate until preset conditions are reached. The calculation model for individuals to update their own position is expressed by the mathematical formula as follows:

(12) $$\vec{D}=\left|\vec{C}\times \vec{P_{rand}}-\vec{P_{i}^{t}}\right|$$

(13) $$\vec{P_{i}^{t+1}}=\vec{P_{rand}}-\vec{A}\times \vec{D}$$

where $\vec{P_{rand}}$ is a randomly generated position vector within the boundary range, $\vec{P_{i}^{t}}$ is the *i* th position vector generation *t* of search agents, and $\vec{P_{i}^{t+1}}$ is the *i* th position vector generation *t*+1 of search agents.

### 3.3. The MOWOA for Solving the Proposed Model

The original WOA is one objective. Kumawat et al. [43] suitably modified the method to solve multi-objective problems, which is known as MOWOA, which has good exploration and exploitation in a given search space. It also has been proven with faster convergence and fewer parameters. MOWOA has been applied to solve many problems. However, MOWOA has still not been applied to solve the optimal offloading mechanism of computation offloading in mobile edge computing. Therefore, in this paper, MOWOA for the optimal offloading mechanism of computation offloading is proposed and adaptively modified to suit our model. Then, the pseudocode of the main procedure of MOWOA is shown in Algorithm 1. The pseudocode of the Non-Dominate sort and crowding degree Sort (ND-C_sort) function is shown in Algorithm 2.


**Algorithm 1: MOWOA**
1: Initialize the whale’s population and set it as Ppopulation2: Hpopulation ← [], set size of Hpopulation as capacity  // Hpopulation represents the external archives3: **While** t < maximum number of iterations **do**4:  Normalize the boundary values when search agents are out of the preset bound value5:  $\left(\mathrm{Hpopulation},\;\vec{P_{*}^{t}})\;=\;\mathrm{ND}-C\_\mathrm{sort}(\mathrm{Hpopulation},\;\mathrm{Ppopulation},\;\mathrm{capacity}\right)$6:  **For** each search agent **do**7:    $\mathrm{Update}\;\vec{a},\;\vec{A},\;\vec{C},\;l\;\mathrm{and}\;w$8:    $\mathrm{If}\;w<0.5\;\mathrm{and}\;\left|\vec{A}\right|\leq 1\;\mathrm{then}$9:      Update the position of the current search agent by using (10)10:    $\mathrm{Else if}\;w<0.5\;\mathrm{and}\;\left|\vec{A}\right|>1\;\mathrm{then}$
11:      Select a random search agent (*X_rand_*)12:      Update the position of the current search agent by using (13)13:    **Else if** w ≥ 0.5 **then**14:      Update the position of the current search by using (11)15:    **End if**16:  **End for**17:  t = t + 118: **End while**19: **Return Hpopulation**


**Algorithm 2: ND-C_sort function**

$$\mathrm{Function}:\;(\mathrm{Hpopulation},\;\vec{P_{*}^{t}})\;=\;\mathrm{ND}-C\_\mathrm{sort}\;(\mathrm{Hpopulation},\;\mathrm{Ppopulation},\;\mathrm{capacity})$$
Input: Hpopulation, Ppopulation, capacity
$$\mathrm{Output}:\;\mathrm{Hpopulation},\;\vec{P_{*}^{t}}$$
1: Add Ppopulation into Hpopulation2: Update Hpopulation with the non-dominated solution of Hpopulation based on formula (6)3: The solutions in Hpopulation are sorted by crowding degree by formula (14)4: **If** size (Hpopulation) > capacity then5:  Solutions that have poor crowding degrees are eliminated6: **End if**7: The optimal solution $\vec{P_{*}^{t}}$ is selected from Hpopulation by the roulette selection method by using (15)

In the beginning, the parameters of MOWOA and computation model are input. First, initialize the whale’s population and set it as Ppopulation of size *m*; Hpopulation is defined as the external archives and the size of Hpopulation is named capacity.

If any target function in F_i_ is not greater than F_j_ and there exists at least one smaller than Fj, it can be regarded as F_i_ dominate F_j_. The decision vector initialized as ranging from zero to one is substituted into formula (6), for solving $F_{1},F_{2},\ldots ,F_{m}$. Additionally, it was merged with the current non-dominated solution set $H^{t-1}$ to be $H^{t}$ by comparing it with the vectors in $H^{t-1}$, where t is the current generations in the iterations of the algorithm. The corresponding decision vector of these target function vectors is also saved. For each solution in $H^{t}$, they will be sorted according to the target function. Then, the crowding degrees of all solutions are calculated based on the formula shown as,

(14) $$d_{i}=\sum_{j=1}^{2}\frac{f_{j}^{i+1}-f_{j}^{i-1}}{f_{j}^{\max}-f_{j}^{\min}}$$

where *i* represents the *i* th solution in $H^{t}$. All solutions in the set $H^{t}$ are sorted in descending order by the crowding degree.

The optimal solution $\vec{P_{*}^{t}}$ is selected from $H^{t}$ by the roulette selection method. The probability for each solution to be chosen can be calculated as,

(15) $$p(F_{i}|H^{t})=\frac{k-i+1}{\sum_{j=1}^{k}j}$$

where $H^{t}$ is the solution set, *k* represents the number of the solution sets. $F_{i}^{}$ is the target function of the *i* th solution.

Then, WOA is used to update the position of the search agents of the next generation based on $\vec{P_{*}^{t}}$.When the preset maximum iteration times are reached, $H^{t}$ is the optimal non-dominated solution set, including the corresponding decision vectors.

### 3.4. The MOWOA2 for Solving the Proposed Model

As the gravity reference point method has the advantages of a better spread of the solution set, the combination of the gravity reference point method with MOWOA can further improve the performance of the computing offloading mechanism in mobile edge computing. In this paper, an algorithm named MOWOA2 by hybridizing the gravity reference point method with MOWOA is proposed. The closer the gravity reference point is, the more attractive it is to the current solution and the greater the weight coefficient is.

In this paper, two objective functions of time and energy consumption are defined. When the time objective function $f_{1}(X)$ takes the maximum, the corresponding solution is called $\vec{X1}$;when the energy objective function $f_{2}(X)$ takes the maximum, the corresponding solution is called $\vec{X2}$. When $X=\vec{X1}$, the value of $f_{1}(X)$ and $f_{2}(X)$ calculated based on fomula (6) is denoted as time1 and energy1 respectively, similarly, when $X=\vec{X2}$, the value of $f_{1}(X)$ and $f_{2}(X)$ calculated based on fomula (6) is denoted as time2 and energy2 respectively. for each solution in solution set H:

- Compute the *time* and *energy* of the current solution, and compute the distance between the current solution and $\vec{X1}$, $\vec{X2}$, which are denoted as dis1 and dis2:
  (16) $$dis1=\sqrt{{\left(time-time1\right)}^{2}+{\left(energy-energy1\right)}^{2}}$$
  (17) $$dis2=\sqrt{{\left(time-time2\right)}^{2}+{\left(energy-energy2\right)}^{2}}$$
- Compute the weight parameter. The nearer gravity reference point has a more attractive force to the current solution, so the weight becomes larger:
  (18) $$\lambda_{1}=\frac{(dis1+dis2)-dis1}{dis1+dis2}=\frac{dis2}{dis1+dis2}$$
  (19) $$\lambda_{2}=\frac{(dis1+dis2)-dis2}{dis1+dis2}=\frac{dis1}{dis1+dis2}$$
- Update the position of the current solution:
  (20) $$\vec{D1}=\left|\vec{C}\times \vec{X1}-\vec{X^{t}}\right|$$
  (21) $$\vec{D2}=\left|\vec{C}\times \vec{X2}-\vec{X^{t}}\right|$$
  (22) $$\vec{X^{t+1}}=\lambda_{1}*\left|\vec{X^{t}}-\vec{A}\times \vec{D1}\right|+\lambda_{2}*\left|\vec{X^{t}}-\vec{A}\times \vec{D2}\right|$$

After all the positions of the solutions are updated, use the non-dominated sort function to update solution set H.

The pseudocode of the Gravity function is shown in Algorithm 3.


**Algorithm 3: Gravity function**
Function: (Tpopulation) = Gravity (Hpopulation, capacity)Input: Hpopulation, capacityOutput: Tpopulation1: Tpopulation ← Hpopulation2: **For** each solution vector of Hpopulation **do**3:   Update the position of the current solution vector by using (22)4:   Return the out-of-bounds solution vector to the boundary5: **End for**6: (Tpopulation, $\vec{X_{*}^{t}}$) = ND-C_sort(Tpopulation, Hpopulation, capacity)

## 4. Numerical Experiments

This section will carry out numerical experiments based on the system model above and the algorithm proposed. A multi-user computation offloading system is simulated, Some useful results are obtained by using MOWOA, MOWOA2, GrEA [44], the Multi-objective Evolutionary Algorithm by Decomposition (MOEA/D-DE) [45], the Multi-objective Evolutionary Algorithm based on Decomposition with Dynamical Re-source Allocation (MOEA/D-DRA) [46], NSGA-III [47], the Epsilon Multi-objective Evolutionary Algorithm (e-MOEA) [48], and CGbAIS [49,50], respectively. Moreover, the algorithms are coded in MATLAB 2016a, and all tests are performed on a PC with a Windows 10 operating system and 8 GB of RAM. The detailed parameters of the multi-user computation offloading system are shown in Table 2.

Moreover, CPU cycles *b* and channel gain *h* are shown as *b* = 0.2*c* and h = 1/*L*^4^.

### 4.1. Performance Indicators

Here are several indicators to evaluate the multi-objective optimization algorithms, such as Generational Distance (GD), convergence metric γ, Spacing, diversity metric ∆, Hypervolume (HV), Inverted Generational Distance (IGD), C-metric and Knee-driven Dissimilarity (KD). The performance of multi-objective algorithms should be judged from the convergence, uniformity and spread of the solution set. The indicator Spacing can evaluate the uniformity of the solution set well, but this indicator does not consider the convergence and spread of the solution set. The indicator HV can both evaluate the convergence and spread of the solution set. The collaborative use of Spacing and HV can cover the assessment criteria overall. Additionally, in most cases, indicators need a real Pareto front to evaluate the performance of a multi-objective optimization algorithm. However, the real Pareto front in practical problems is hard to acquire. Fortunately, both Spacing and HV do not need to have a known Pareto Front, which fits our requirement exactly. Therefore, two indicators are used as metrics: Spacing [51] and HV [52]. The Spacing metric can be used to measure the uniformity of the solution set of multi-objective algorithms, especially in the two-dimensional case (which fits nicely with this article). If Spacing is smaller, it means better uniformity of the solution set. The larger the HV, the better the performance of convergence and spread of the solution set. Their definitions are denoted as:

(23) $$Spacing=\sum_{i=1}^{\left|PF\right|}\frac{d_{i}-\bar{d}}{\left|PF\right|}$$

(24) $$HV=\delta \left(\underset{x\in S}{\cup }\left[f_{1}\left(x\right),r_{1}^{*}\right]\times \left[f_{2}\left(x\right),r_{2}^{*}\right]\right)$$

### 4.2. Numerical Results and Analysis

In this section, the detailed experimental results are reported. To investigate the performance of each algorithm in different situations, the number of users is set to 30, 45, and 60, respectively.

To better examine the MOWOA proposed, the result of our method is compared with that of GrEA, MOEA/D-DE, MOEA/D-DRA, NSGA-III, e-MOEA, and CGbAIS. The iteration times of all algorithms is set to 30. The quantity of search agents of MOWOA and MOWOA2 is 100. The capacity of external archives of MOWOA and MOWOA2 is 30. Correspondingly, the amount of search agents of GrEA, NSGA-III, and e-MOEA is 30, and all these three algorithms are employed with real number coding. For MOEA/D-DE and MOEA/D-DRA, the probability that parent solutions are selected from the neighborhood is 0.9. Additionally, the maximal number of solutions replaced by a child solution is two. Both MOEA/D-DE and MOEA/D-DRA are employed with the penalty-based boundary intersection (PBI) [53] to disintegrate. As for CGbAIS, population size is set as 100; memory capacity is set as 30; crossover probability is set as 0.4; the diversity evaluation parameter is set as 0.95; the number of iterations is set as 30.

The different performances of these algorithms when the user number is 30, 45, and 60, respectively, are shown in Table 3, Table 4 and Table 5. To be specific, it includes min/mean time and energy required to obtain the result and the Spacing and HV of the solution set. It should note that when the user number is 30, 45, and 60, respectively, the reference point of computing HV is (1.28, 450), (1.84, 900), and (2.38, 800), respectively. The mean values of time and energy consumption are obtained by performing 100 experiments.

From Table 3, MOWOA2 has a better result than the other algorithms from analysis of the time and energy consumption, which is 1.1918 for the shortest time and 4.6888 × 10^−18^ for the lowest energy. This value has a huge difference in magnitudes from the results of other algorithms. Table 3 and Table 4 represent that the lowest energy consumption and average energy consumption of MOWOA are better than the other algorithms except for MOWOA2, while MOWOA2 has the best time and energy consumption.

Additionally, an interesting phenomenon is found by comparing Table 3, Table 4 and Table 5. As the user number increases, the time consumption has the tendency to increase; however, the energy consumption seems not to obey the rule. In particular, the minimum and mean energy consumption are 13.9848 and 161.6287, respectively, when the user number is 60. While the values are 32.3088 and 261.5592, respectively, when the user number is 45 can be seen. It can be supposed that the energy consumption is not a linear relation with the increase in user number.

It can be shown from Table 3, Table 4 and Table 5 that when user number is 30, 45, and 60, the HV value of the MOWOA is 28.6767, 104.9165, and 132.8395, higher than other algorithms except MOWOA2. It means the MOWOA has better convergence and multiformity than other algorithms. Moreover, Table 3, Table 4 and Table 5 represent the HV value of MOWOA2 as 35.3101, 122.7615, and 132.9202, respectively, in the corresponding situation. That means that the improved whale optimization algorithm, MOWOA2, will further exploit its advantages, and its overall performance is much better than the other algorithms. Additionally, it can be seen that the HV index of the MOEA/D-DE, MOEA/D-DRA and e-MOEA algorithms is very poor, all of which rank at the bottom in all three cases. The reason is that these three algorithms based on decomposition strategy can achieve good results by decomposing the optimization of many objectives, which shows that the convergence and diversity of the solution sets obtained by them are not good. It suggests that the decomposition strategy is not appropriate for this problem.

Then, it can be seen from Table 3, Table 4 and Table 5 that, when the number of users is 30 and 45, the Spacing value of the solution set of e-MOEA is the best, with values of 0.0375 and 0.0129, respectively. When the number of users is 60, the Spacing index for the MOEA/D-DE solution set is the best, at 0.0352. This shows that the two algorithms perform well in the aspect of uniformity. In these three cases, the Spacing indexes of the CGbAIS solution set are 4.7710, 2.9393 and 0.8152, respectively, which are almost all the worst. This indicates that the uniformity of CGbAIS is not good. Considering the structure of the CGbAIS algorithm, this algorithm adopts the maximum and minimum distance method, which can group the solutions based on distance. Then, some solutions with near distance are deleted from grouped solutions and only one solution is reserved, to simplify the solution set. However, this also leads to a decrease in uniformity. When the number of users is 30, MOWOA2 and MOWOA are not too bad for uniformity, with Spacing of 0.1658 and 0.2797, respectively, ranking third and fourth among the eight algorithms. However, as the user size increases, they tend to show worse uniformity. With the increase in the number of users, the space of the feasible domain increases, resulting in a larger distance between the solutions obtained by the MOWOA2 and MOWOA. Therefore, the Spacing indexes obtained by the two algorithms are higher than those of other algorithms.

In order to make the conclusion more intuitive and convincing, statistical methods were introduced. Single-sample *t*-tests were used to examine significant differences be-tween the Spacing and HV of MOWOA2 and other algorithm metrics. As performing single-sample *t*-test needs to meet normality distribution, the normality test of the sample was per-formed on all the other algorithms indexes except MOWOA2, which was used as a reference value. When the number of samples is small, the results of normality test should be measured by using the Kolmogorov–Smirnov (K-S) index. The results of normality tests are shown in Table 6.

As shown in Table 6, Sig values are 0.2 and 0.174, both of which are larger than 0.05. Therefore, they obey normal distribution, which meets the condition of a single sample *t*-test. Furthermore, the Sig value of Spacing is 0.001 < 0.05 when the number of users is 30, which does not conform to the normal distribution, so the single sample *t*-test cannot be used.

The single-sample *t*-test was then used to examine the significant differences, with 95% confidence intervals selected by default. As can be seen from Table 7, all Sig.(two-tailed) values of all test results for these indicators are less than 0.05, so it is considered that the test value is significantly different from the sample mean. The Spacing and HV indexes of MOWOA2 are significantly different from those of other algorithms.

**Figure 3:**
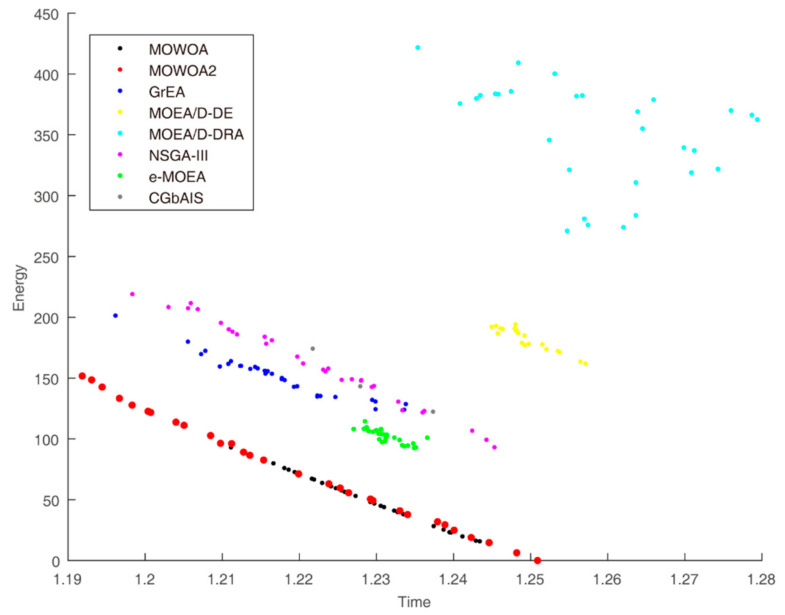
Time and energy consumption of the different algorithms when the user number is 30.

**Figure 4:**
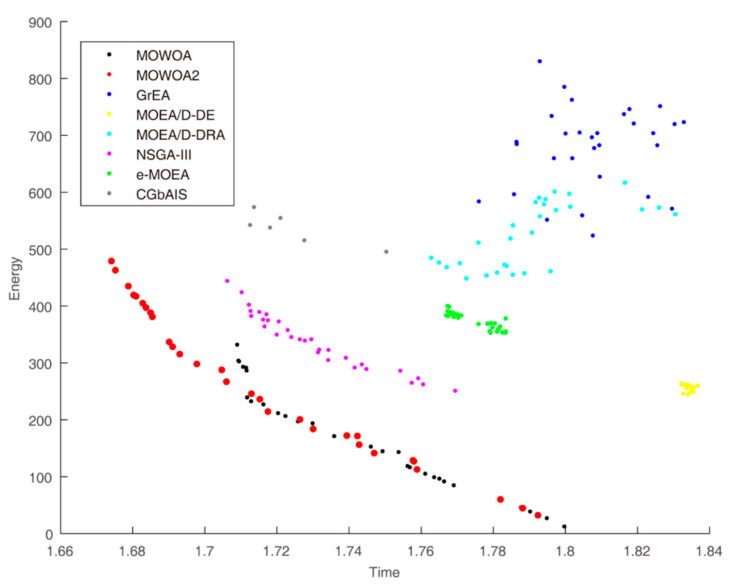
Time and energy consumption of the different algorithms when the user number is 45.

**Figure 5:**
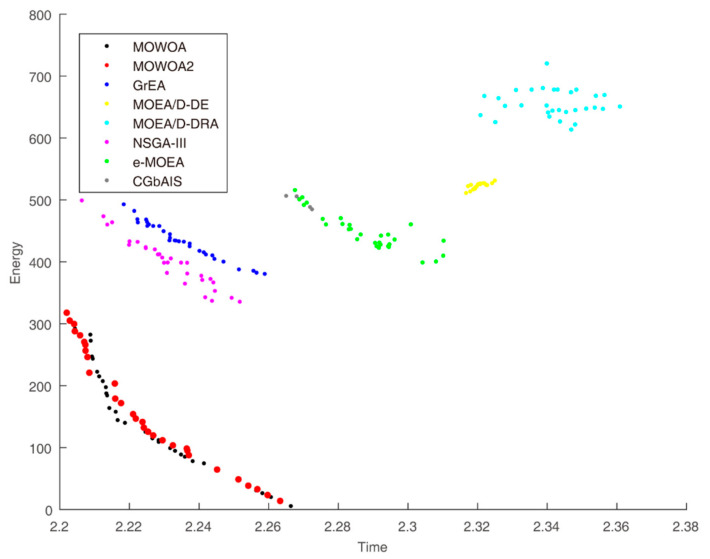
Time and energy consumption of the different algorithms when the user number is 60.

Time and energy consumption obtained by the different algorithms when the user number is 30, 45 and 60 are shown in Figure 3, Figure 4 and Figure 5, respectively. They indicate the differences in convergence and diversity among different algorithms. Compared with the solution sets of the other algorithms, the solution sets of both MOWOA and MOWOA2 are distributed on the lower left in the three Figures, which show that MOWOA and MOWOA2 are closer to the Pareto optimal front. Therefore, the two algorithms proposed in this paper have the advantages in convergence and diversity. In addition, in terms of convergence, the two algorithms perform equally and, in terms of diversity, the position of a redpoint is near to the abscissa axis in Figure 3, Figure 4 and Figure 5, which indicates MOWOA2 improved by using the gravity reference point method can enhance the ability of exploration. Therefore, MOWOA2 is better than MOWOA.

From Figure 3, it can be seen that the shape of the solution sets obtained by these algorithms in the target function space are different. The shape of the solution sets of MOEA/D-DRA resembles a cloud, which means the convergence of the algorithm is the worst of all algorithms. The lines of the solution sets of MOWOA and MOWOA2 are apparently much closer to the Pareto Front. In addition, although the graphs obtained by other algorithms appear linear, they are far away from the Pareto Front, indicating that their solution set is not optimal.

From Figure 4, the shape of the solution sets of both GrEA and MOEA/D-DRA are divergent, which shows the convergence of the algorithms is worse than that of other algorithms. The lines of the solution sets of MOWOA and MOWOA2 are apparently much closer to the Pareto Front. Although the shape of the solution set obtained by NSGA-III is also linear, it is inferior to our proposed algorithms. Moreover, the graphs obtained by CGbAIS, e-MOEA and MOEA/D-DE appear relatively concentrated, which indicates that the diversity is poor, and it is far away from Pareto Front, which also indicates that the convergence is poor.

From Figure 5, the shape of the solution sets of MOEA/D-DRA and e-MOEA are slightly divergent and far away from Pareto Front when the user number is 60, which shows that the convergence of the algorithms is worse than that of other algorithms. The lines of the solution sets of MOWOA and MOWOA2 are apparently much closer to the Pareto Front. Although the shape of the solution set obtained by NSGA-III is also linear, it is inferior to our proposed algorithms. Furthermore, the graphs obtained by CGbAIS, e-MOEA and MOEA/D-DE appear relatively concentrated, which indicates that the diversity is poor, and it is, which also indicates that the convergence is poor.

**Figure 6:**
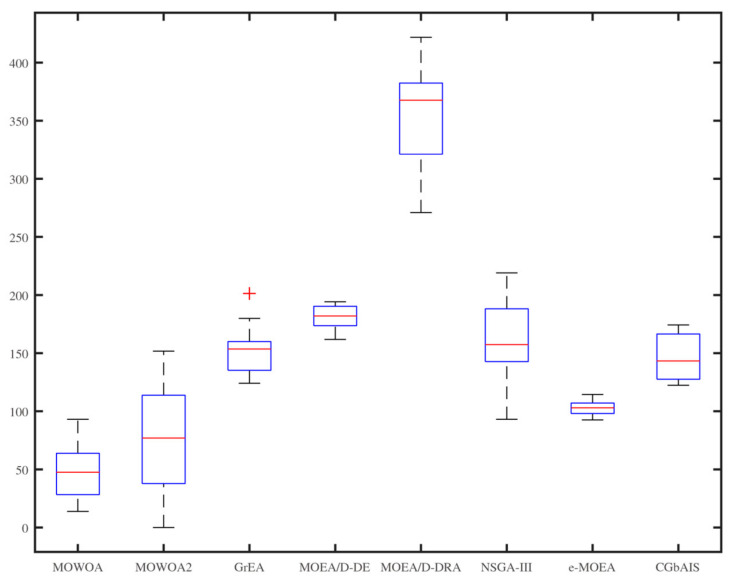
Energy consumption of the different algorithms when the user number is 30.

**Figure 7:**
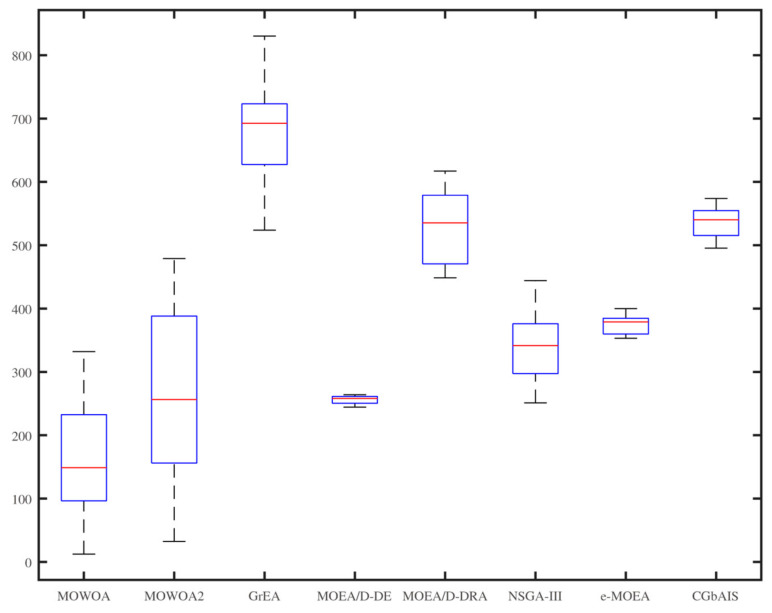
Energy consumption of the different algorithms when the user number is 45.

**Figure 8:**
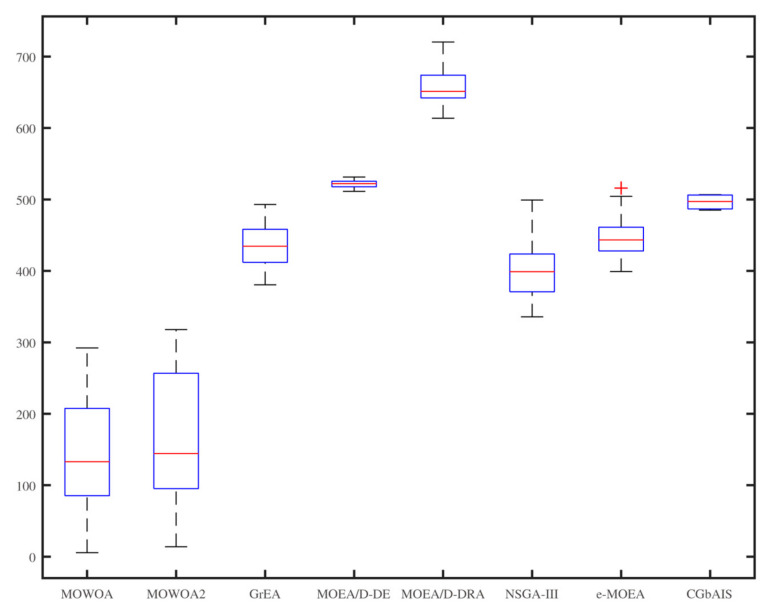
Energy consumption of the different algorithms when the user number is 60.

**Figure 9:**
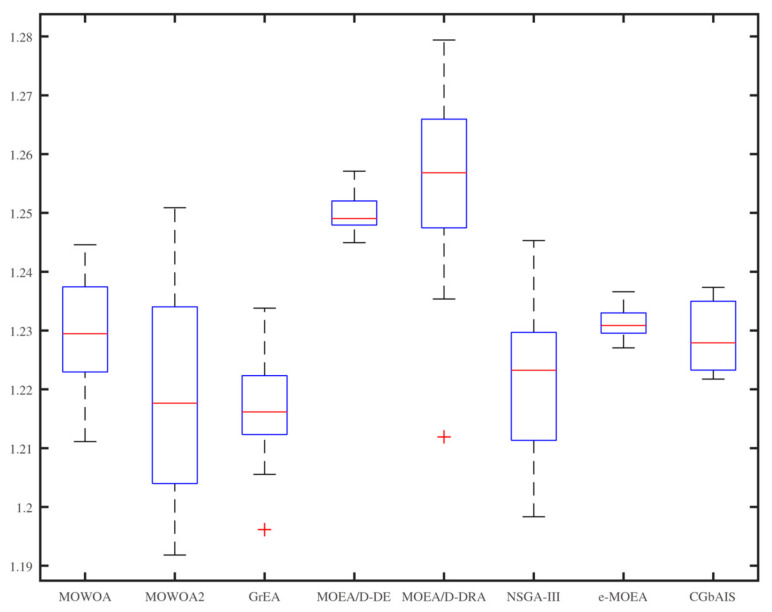
Time consumption of the different algorithms when the user number is 30.

**Figure 10:**
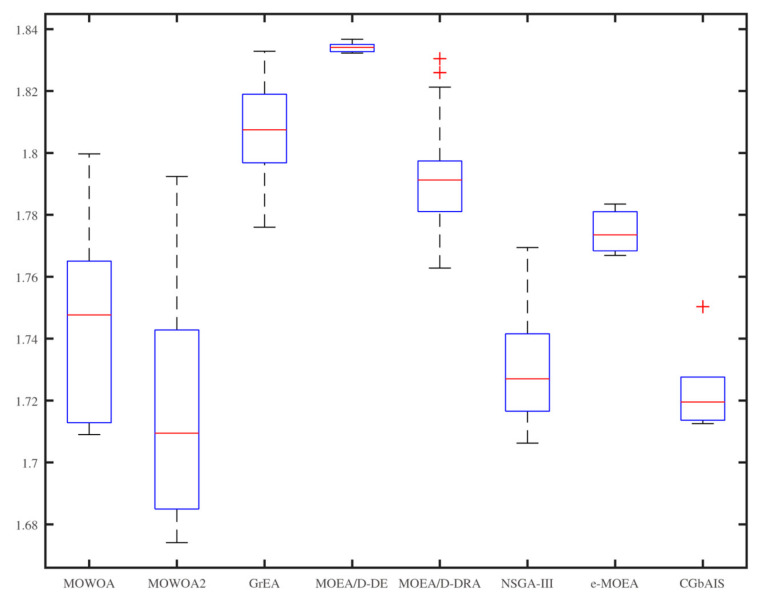
Time consumption of the different algorithms when the user number is 45.

**Figure 11:**
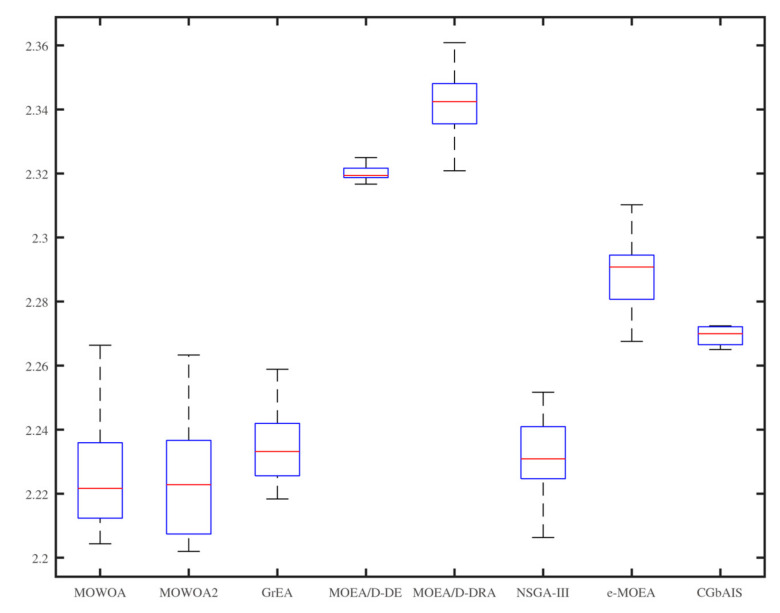
Time consumption of the different algorithms when the user number is 60.

From Figure 6, Figure 7, Figure 8, Figure 9, Figure 10 and Figure 11, the box figures of the solution sets of energy and time consumption are shown when the user number is 30, 45, and 60, respectively. The red line is the average energy consumption of different solution sets by different algorithms. The diversity of solution sets can be objectively judged according to the length of the blue rectangles. The box figures of energy consumption obtained by different algorithms for different numbers of users can be seen from Figure 6, Figure 7 and Figure 8, The box figures of time consumption obtained by different algorithms for different numbers of users can be seen from Figure 9, Figure 10 and Figure 11.

The average energy consumptions of the solution sets obtained by MOWOA and MOWOA2 are obviously lower than that obtained by other algorithms. The average time consumption of the solution sets obtained by MOWOA2 is obviously lower than that obtained by other algorithms.

The length of the rectangles obtained by MOWOA2 is obviously longer than that obtained by other algorithms, which indicates the diversity of the solution sets of MOWOA2 is excellent. In particular, the length of the rectangles obtained by MOWOA2 is longer than that of MOWOA, which means the diversity of MOWOA2 is better than MOWOA when applying the gravity reference point method.

In Figure 6, it can be seen that the average energy consumption of the solution sets of MOWOA is the lowest, and that of MOWOA2 is the second-lowest when the user number is 30. However, it does not show that the average energy consumption of the solution sets obtained by MOWOA2 is worse than that of MOWOA. In fact, it can be seen from Figure 9 that the average time consumption of the solution sets of MOWOA2 is the lower than that of MOWOA when the user number is 30. As mentioned in the previous part of the article, time and energy consumption are mutually exclusive targets. The solution set of MOWOA2 trades higher energy consumption for lower time consumption. 

In Figure 6 and Figure 9, although the time consumption of GrEA is slightly lower than that of MOWOA2 when the number of users is 30, the energy consumption of GrEA is much higher than that of MOWOA2.

In Figure 8 and Figure 11, When the user number is 60, it shows that the average time and energy consumption of the solution sets of MOWOA and MOWOA2 are almost the same, both of which are better than other algorithms. 

It can be observed from these figures that time consumption and energy consumption of the solution sets obtained by MOEA/D-DE, MOEA/D-DRA, GrEA, e-MOEA, NSGA-III, and CGbAIS are worse than that obtained by MOWOA and MOWOA2 under the three different settings. 

To sum up, we can conclude that MOWOA2 applied to computation offloading mechanism optimization has excellent performance on convergence and diversity.

## 5. Discussion

In this paper, MOWOA based on the non-dominated sort is applied to solve the computational offloading model. Then, in order to further improve the extensiveness of the solution set, the gravity reference point method is proposed and applied to improve MOWOA, which is named MOWOA2. The box charts show that the extensiveness of the MOWOA2 algorithm is better than that of MOWOA, and it is also better than the other six algorithms.

Furthermore, the experience results show that the energy consumption of MOWOA and MOWOA2 are significantly lower than that of other algorithms. The time consumption is only slightly lower than other algorithms. This is because time and energy consumption are mutually exclusive. An explanation of this can be drawn from the figures and tables. For example, it can be seen from Table 3 that the energy consumption of MOWOA2 is higher than that of MOWOA while its time consumption is lower than that of MOWOA. As seen from Figure 4, MOWOA and MOWOA2 still are in the same Pareto Front, though the distribution of MOWOA2 is closer to the vertical axis. This indicates that the convergence of the two algorithms is the same; the different distribution of the solution sets lies in the fact that MOWOA2 increases the diversity of solution sets by introducing the gravity reference points method. In addition, it can be seen from the figures that the solution sets of MOWOA and MOWOA2 are closer to the origin, indicating that they are closer to the true Pareto Front. This proves that the convergence of MOWOA and MOWOA2 is better than other algorithms. In addition, the HV Index of MOWOA and MOWOA2 is significantly different from that of other algorithms. Higher HV values indicate that the two algorithms have excellent comprehensive performance, including convergence and diversity.

It is a pity that the uniformity of MOWOA and MOWOA2 measured by the Spacing metric is inferior to other algorithms. Finally, in order to obtain the most appropriate number of search agents for MOWOA2, the algorithm was tested with the number of search agents as 50, 80, 100, 120, and 150 when the number of users was 30, 45, and 60, respectively. The minimum and mean time consumption, the minimum and mean energy consumption and the average running time of MOWOA2 under these conditions were obtained, as shown in Table 8.

It can be seen from Table 8, with the increase in the number of search agents, that the minimum and mean time and energy consumption decrease. That is, the convergence performance of the algorithm is gradually enhanced. This is because the number of solutions generated by the algorithm increases, which guarantees that the algorithm will explore more possible solutions. However, when the number of search agents is 100, the trend of decline is no longer obvious. When the number of search agents reached 150, the algorithm’s minimum and mean energy consumption rebounded in all three cases, while it time consumption was only slightly reduced. Most importantly, as the number of search agents increases, so does the average run-time of the algorithm. This means that blindly increasing the number of search agents in pursuit of the improvement of convergence performance is not a good choice because it will lead to a longer running time of the algorithm. Therefore, a compromise was chosen—the number of search agents was set to 100.

## 6. Conclusions

In this paper, we apply MOWOA to solve the optimal offloading mechanism of the computation offloading in mobile edge computing. Since the extensiveness of the solution set of MOWOA on this problem is not good enough, the gravity reference point method is proposed to improve it, which is named MOWOA2. Under different numbers of users, we compare the performance of MOWOA2 with some other classical optimization algorithms. The experimental results show MOWOA2 is high-performance, showing high convergence, good diversity, and low complexity. However, it can be found that the uniformity of MOWOA2 is not good enough. In the future, we will continue to make improvements to MOWOA2 to overcome this shortcoming.

As a matter of fact, the real scenario of edge computing is much more complicated. Therefore, how to broaden the practicability of the algorithm in the complex communication and offloading environment still has a long way to go. There are still many factors we need to consider. In the future, we will try to apply other intelligent algorithms to the computation offloading problem. Moreover, we will conduct further research on continuing to improve the performance of MOWOA and apply this algorithm to other areas.

## 7. Patents

202010497910.0, an allocation method for computation offloading of edge servers, Huang Mengxing, Zhai Qianhao, Feng Siling, Luo zaici, Deng Yang. 4 June 2020.

## Figures and Tables

**Table 1:** The advantages and weakness of the works mentioned.

Reference	Methodology	Advantages	Weakness
[13]	Game-theoretic	Multi-user; practical	Has no consideration on energy
[14]	Game-theoretic	Hybrid with dynamic voltage scaling	Separately considers energy minimum and latency minimum
[15]	Game-theoretic	Energy-aware; light	Solutions not significant
[16]	Game-theoretic	Using online learning in the iterative algorithm	Hard to implement
[17]	Game-theoretic	Multi-user; multi-task; secure	Specialized application
[18]	Game-theoretic	Multi-user; low time complexity	Only considers time overhead
[19]	Game-theoretic	For pervasive scenario; innovative	Hard to implement
[20]	Machine learning	Multi-user; a simple procedure	Not suitable for complex network
[21]	Machine learning	Multi-server; Multi-user; Multi-task	Binary offloading
[22]	Machine learning	Conducts a failure recovery policy	Low convergence
[23]	Machine learning	Partitioning to subtasks; parallel offloading	High complexity
[24]	Machine learning	Fully distributed	No synchronization between mobile user and edge server
[25]	Machine learning	Optimal offloading solution	High complexity; low convergence for real-time application

**Table 2:** Detailed parameters of the multi-user computation offloading system.

Symbol	Description	Value
c	size of data	4.5 × 10^4^–5.0 × 10^4^ kB
P	maximum transmission power	10–100 W
$f_{l}$	computation capacity of local devices	0.5–1 GHz
$f_{c}$	computation capacity of edge servers	10 GHz
L	distance between local devices and edge servers	1–30 m
ω	channel bandwidth	5.0 × 10^−3^ GHz
$\sigma^{2}$	background noise power	1.0 × 10^−13^ w

**Table 3:** The values of indicators using different algorithms when the user number is 30. The bold number is the best value in this column.

Algorithms/Indicators	Time		Energy		Spacing	HV
	Min	Mean	Min	Mean		
MOWOA2	**1.1918**	1.2193	**4.6888 × 10^−18^**	76.0509	0.1658	35.3101
MOWOA	1.2111	1.2295	13.8006	**48.0954**	0.2797	28.6767
GrEA	1.1961	**1.2173**	124.0556	151.2140	0.3128	25.9536
MOEA/D-DE	1.2449	1.2499	161.8432	181.1392	0.0552	9.9063
MOEA/D-DRA	1.2119	1.2569	270.9970	352.4629	0.3245	6.9231
NSGA-III	1.1983	1.2220	93.1188	161.1363	0.3042	25.7863
e-MOEA	1.2270	1.2312	92.5499	102.4075	**0.0375**	18.8615
CGbAIS	1.2217	1.2289	122.3683	146.6156	4.7710	18.5727

**Table 4:** The values of indicators using different algorithms when the user number is 45. The bold number is the best value in this column.

Algorithms/Indicators	Time		Energy		Spacing	HV
	Min	Mean	Min	Mean		
MOWOA2	**1.6741**	**1.7173**	32.3088	261.5592	1.7120	122.7615
MOWOA	1.7090	1.7449	**12.4587**	**163.7678**	0.7835	104.9165
GrEA	1.7760	1.8074	523.8285	678.7592	1.2088	25.9536
MOEA/D-DE	1.8322	1.8341	244.5273	256.1590	0.0475	5.0817
MOEA/D-DRA	1.7628	1.7915	448.4761	528.7081	0.7596	34.6198
NSGA-III	1.7062	1.7303	251.1581	339.2763	0.6388	82.3174
e-MOEA	1.7669	1.7747	353.0988	374.1175	**0.0129**	19.6814
CGbAIS	1.7125	1.7238	495.4125	536.6364	2.9393	50.4503

**Table 5:** The values of indicators using different algorithms when the user number is 60. The bold number is the best value in this column.

Algorithms/Indicators	Time		Energy		Spacing	HV
	Min	Mean	Min	Mean		
MOWOA2	**2.2020**	**2.2251**	13.9848	161.6287	0.8601	132.9202
MOWOA	2.2043	2.2268	**5.7005**	**141.6998**	0.5095	132.8395
GrEA	2.2184	2.2352	380.6338	434.3730	0.2987	65.8733
MOEA/D-DE	2.3167	2.3200	511.3582	521.6261	**0.0352**	18.2733
MOEA/D-DRA	2.3208	2.3416	613.6902	655.5079	0.4611	10.6627
NSGA-III	2.2063	2.2313	335.7004	400.5358	0.2607	77.3336
e-MOEA	2.2675	2.2880	399.0791	448.6972	0.0882	43.1851
CGbAIS	2.2650	2.2693	484.9868	496.5318	0.8152	36.0765

**Table 6:** Normality Test of samples used by all other algorithms except the Multi-Objective Whale Optimization Algorithm (MOWOA2).

	Kolmogorov–Smirnov
	Sig.
**Spacing30**	0.001
**HV30**	0.200
**Spacing45**	0.200
**HV45**	0.174
**Spacing60**	0.200
**HV60**	0.200

**Table 7:** The single-sample *t*-test of MOWOA2.

	Sig. (2-Tailed);
**Spacing** **30**	/
**HV30**	0.008
**Spacing45**	0.002
**HV45**	0.005
**Spacing60**	0.001
**HV60**	0.009

**Table 8:** The results of adjusting parameters.

The Number of Users	Number of Search Agents	Time		Energy		Average Running Time
		Min	Mean	Min	Mean	
30	50	1.1782	1.2062	19.2116	68.5468	0.2259s
	80	1.1723	1.2025	18.9858	64.4145	0.2925s
	100	1.1690	1.2005	18.2421	63.1191	0.3470s
	120	1.1687	1.2003	18.4659	63.3787	0.4033s
	150	1.1684	1.1997	18.8542	64.5663	0.4927s
45	50	1.7108	1.7473	30.5254	250.4384	0.2278s
	80	1.7050	1.7419	28.4220	248.0583	0.3120s
	100	1.7002	1.7400	27.3841	248.3327	0.3608s
	120	1.6980	1.7391	27.2972	248.8614	0.4267s
	150	1.6979	1.7388	27.6336	249.5070	0.5292s
60	50	2.2138	2.2351	15.5345	163.3253	0.2648s
	80	2.2059	2.2323	14.9360	161.8343	0.3515s
	100	2.2016	2.2284	14.6777	157.6920	0.4045s
	120	2.2011	2.2235	14.4166	157.5522	0.4659s
	150	2.2008	2.2223	14.8116	158.1516	0.5535s

## Data Availability

Data sharing not applicable.
